# Coverage-Conflict-Aware RFID Reader Placement with Range Adjustment for Complete Tag Coverage in IIoT †

**DOI:** 10.3390/s25237400

**Published:** 2025-12-04

**Authors:** Chien-Fu Cheng, Bo-Yan Liao

**Affiliations:** 1 Department of Computer Science and Engineering, National Taiwan Ocean University, Keelung 202301, Taiwan; 2 Department of Computer Science and Information Engineering, Tamkang University, New Taipei 251301, Taiwan; 607420030@s07.tku.edu.tw

**Keywords:** radio frequency identification (RFID), industrial internet of things (IIoT), reader-to-tag interference, reader-to-reader interference, collision avoidance, deterministic reader deployment, adjustable reading range, tag coverage optimization

## Abstract

Radio Frequency Identification (RFID) is a core enabler of the Industrial Internet of Things (IIoT), yet dense deployments suffer from tag collisions and reader interference that degrade reliability and inflate infrastructure cost. This study proposes a deterministic Reader Deployment Algorithm with Adjustable Reader range (RDA2R) to achieve full tag coverage with minimal interference and reader usage. The method divides the monitored field into grid units, evaluates tag coverage weights, activates high-weight readers with interference checks, and adaptively adjusts interrogation ranges. Simulation results under random and congregation tag distributions show that RDA2R requires about 46–47% fewer readers than ARLDL and 32–33% fewer than MR2D, while improving average tag coverage per reader by over 30%. These results demonstrate that RDA2R provides a scalable, interference-aware, and cost-efficient deployment strategy for RFID-enabled IIoT environments.

## 1. Introduction

The rapid evolution of Wireless Sensor Networks (WSNs) has laid the foundation for pervasive data acquisition in large-scale environments. A typical WSN integrates a multitude of miniature sensing devices that possess sensing, computation, and communication capabilities. These nodes are cost-effective and capable of measuring diverse physical parameters such as temperature, humidity, and pressure, which makes them suitable for deployment in military, agricultural, industrial, and healthcare applications [1,2,3,4]. The convergence of such sensor-based networks with Internet connectivity has accelerated the formation of the Internet of Things (IoT), a paradigm that enables continuous monitoring and autonomous control of physical assets through distributed sensing and communication [5,6,7]. Applications of IoT have become widespread and include smart infrastructure, intelligent transportation systems, and remote healthcare monitoring.

Building upon the foundation of IoT, industrial domains have increasingly integrated artificial intelligence (AI), edge computing, and big data analytics into their cyber-physical infrastructures [8,9,10]. These technological advances have given rise to the Industrial Internet of Things (IIoT), where large-scale sensor networks and machine-to-machine communication enable automation, predictive maintenance, and real-time operational decision-making [11,12,13]. As factories, warehouses, and logistics hubs continue to adopt IIoT solutions, ensuring dependable data acquisition has become essential for safe and efficient operations.

Among the various identification mechanisms used in IIoT, Radio Frequency Identification (RFID) has emerged as a critical technology for object tracking, material flow monitoring, and inventory automation [14,15]. RFID enables non-line-of-sight and high-speed identification, making it suitable for dense industrial deployments. However, large-scale RFID systems are highly susceptible to interference problems such as tag collision, reader-to-reader interference, and reader-to-tag interference, which severely degrade communication reliability and create coverage gaps [16].

These interference problems are largely determined by the spatial arrangement of readers and the configuration of their interrogation ranges. When readers are placed too closely or operate with unnecessarily large interrogation radii, overlapping interrogation zones may create severe conflicts that result in missed readings, increased latency, and unpredictable system behavior. Consequently, designing a reader deployment strategy that simultaneously guarantees complete tag coverage and minimizes interference is crucial for dependable IIoT operation.

Although prior studies have proposed communication-level protocols or post-deployment optimization methods to alleviate interference, most rely on random or heuristic initialization strategies. These approaches may reduce redundant readers but cannot guarantee collision-free deployment or complete tag coverage, especially in dense or non-uniform environments.

Motivated by these challenges, this study proposes a deterministic reader-deployment framework designed to ensure collision-free coverage and dependable identification in RFID-enabled IIoT systems. Unlike traditional random or heuristic strategies, the proposed approach eliminates randomness in the decision-making process and provides stable and interference-resilient deployment outcomes. Such determinism is especially important in IIoT environments, where industrial automation requires consistent identification quality, minimal signal conflicts, and reliable system operation. To achieve this, the algorithm incorporates four key design principles.

1. Grid-based initialization: The monitored area is divided into uniform grid units to systematically enumerate all potential reader locations, avoiding suboptimal placements caused by random sampling.
2. Weighted tag assignment: Each virtual reader is assigned a coverage weight using a competitive nearest-distance rule, creating an importance-ordered list that prioritizes the most beneficial deployment positions.
3. Interference-aware selection: A candidate reader is activated only if it introduces no reader-to-tag or reader-to-reader conflict with previously deployed readers, ensuring reliable and interference-free operation.
4. Iterative range adjustment: After placement, each deployed reader refines its interrogation radius to the distance of its farthest assigned tag, minimizing redundant overlap and enabling additional non-conflicting deployment opportunities.

By integrating these principles, the proposed algorithm achieves complete tag coverage with fewer readers and effectively mitigates both reader-to-tag and reader-to-reader interference, thereby enhancing the overall reliability and efficiency of RFID-based IIoT environments. This article is a revised and expanded version of a paper entitled “An Enhanced Approach for RFID Reader Deployment in Industrial IoT Systems with Collision Avoidance and Optimized Coverage,” which was presented at the 10th IEEE International Conference on Dependable Systems and Their Applications (IEEE DSA 2023), Tokyo, Japan, 10–11 August 2023 [17].

The remainder of this paper is organized as follows. Section 2 reviews related research on RFID interference mitigation and reader deployment. Section 3 presents the system model and formulates the deterministic deployment problem. Section 4 describes the proposed RDA2R algorithm. Section 5 analyzes computational complexity. Section 6 evaluates performance, and Section 7 concludes the study.

## 2. Related Work

**Figure 1:**
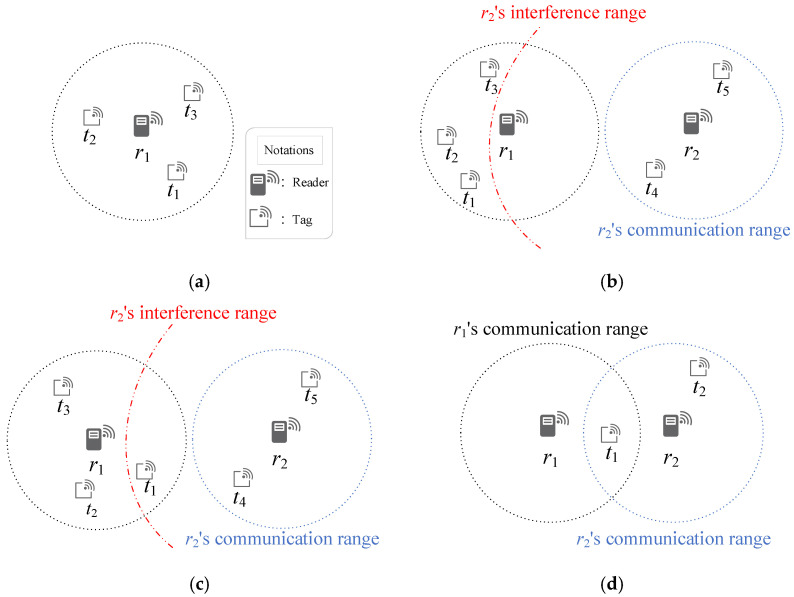
Illustrations of interference scenarios in RFID systems: (**a**) tag collision, (**b**) reader-to-reader interference type 1, (**c**) reader-to-reader interference type 2, and (**d**) reader-to-tag interference.

In RFID systems, communication reliability is frequently hindered by two major types of interference: tag collisions and reader collisions. Before reviewing existing solutions, Figure 1 illustrates these interference scenarios and highlights their spatial complexity in large-scale IIoT deployments. In Figure 1a, tag collisions occur when multiple tags attempt to communicate with the same reader simultaneously, leading to reception failure. Figure 1b,c present two forms of reader-to-reader interference: in Figure 1b, overlapping interference ranges of two readers disrupt one another’s signals, whereas in Figure 1c, one reader’s interference range overlaps with the communication path between another reader and a tag, causing data loss. In addition, Figure 1d depicts reader-to-tag interference, where a tag located within the overlapping interrogation zones of multiple readers undergoes simultaneous interrogation and thus communication failure. These examples underscore the diverse interference patterns that arise in dense RFID deployments. Accordingly, the following section reviews representative approaches proposed to mitigate these two major categories of interference in practical RFID systems.

### 2.1. Tag Collision

A considerable body of research has focused on minimizing tag collisions by regulating the active response time of tags. Among the most prominent approaches is the Dynamic Framed Slotted ALOHA (DFSA [18]). The DFSA [18] protocol dynamically adjusts the frame length according to the estimated number of tags within the interrogation field. By observing the number of collision slots, the system estimates tag population and tunes the frame size accordingly to improve identification efficiency.

Subsequent studies have refined this idea to enhance responsiveness. For example, the Improved Linearized Combinatorial Model (ILCM [19]) emphasized the need for a faster estimation of the number of responding tags to further reduce idle and collision slots. The authors of ILCM [19] proposed an adaptive frame adjustment mechanism that improves DFSA [18] efficiency. However, they also observed that conventional tag estimation methods are computationally intensive, leading to latency during estimation. To address this, ILCM [19] enables more efficient tag estimation while maintaining low computational cost.

### 2.2. Reader Collision

Reader collision is typically divided into two categories: reader-to-reader interference and reader-to-tag interference. The following subsections summarize research efforts addressing each category.

#### 2.2.1. Reader-to-Reader Interference

Reader-to-reader interference occurs when the transmission signals of multiple readers overlap in frequency or time, resulting in degraded signal quality and packet loss. To mitigate this issue, several distributed power control and coordination mechanisms have been proposed. DPC [20] introduces a power-control–based conflict avoidance scheme that dynamically adjusts the transmission power of readers to minimize the overlap between their interrogation ranges. Similarly, CSMA [21] has been explored as a coordination mechanism, allowing readers to sense channel activity and schedule interrogation timing to reduce mutual interference.

#### 2.2.2. Reader-to-Tag Interference

Reader-to-tag interference represents a more intricate problem, as it involves multiple readers simultaneously attempting to communicate with the same tag. Existing studies have approached this challenge through two main strategies: redundant reader elimination and deterministic deployment planning.

The first strategy aims to reduce the number of active readers while maintaining adequate coverage. Neighboring Coverage Density (NCD [22]) allows each reader to assign weights to nearby tags according to their spatial density, and redundant readers are then deactivated based on these weights to reduce interference. An enhanced version, Neighboring Coverage Density Movement Detection (NCDMD [22]), further incorporates motion detection to minimize unnecessary tag write operations in dynamic environments.

Threshold Selection Algorithm (TSA [23]) determines the activation of readers according to a descending order of coverage thresholds. To accommodate tag mobility, the TSAMD (Threshold Selection with Movement Detection) [23] extension dynamically re-evaluates threshold values when tag positions change. Similarly, the Dynamic Range-Based Algorithm (DRBA [24]) iteratively selects readers maximizing tag coverage and adjusts their reading ranges in a recursive manner until full coverage is achieved or no further readers can be activated.

Although redundant-reader–elimination methods can reduce interference and lower reader density, they often depend on randomly initialized deployments and cannot ensure full tag coverage. Deterministic deployment approaches, on the other hand, are more suitable when complete coverage and stable communication performance are required. A representative method is the Minimum-Cost RFID Reader Deployment algorithm (MR2D [25]), which formulates reader placement as a deterministic optimization problem using known tag coordinates and a predefined set of allowable reading ranges. By clustering spatially close tags and assigning readers accordingly, MR2D [25] identifies deployment positions that achieve full coverage while minimizing cost and preventing reader-to-tag interference.

To clarify the strengths and limitations of representative methods, Table 1 summarizes seven approaches: DFSA [18], ILCM [19], DPC [20], CSMA [21], NCD [22], NCDMD [22], TSA [23], DRBA [24], and MR2D [25]. DFSA [18] and ILCM [19] reduce tag collisions through dynamic frame adjustment and improved tag-population estimation, respectively, but neither addresses interference between readers. DPC [20] adapts transmission power to manage reader interference, and CSMA [21] relies on carrier sensing, although both operate only after deployment and cannot ensure collision-free coverage in dense environments. NCD [22], NCDMD [22], TSA [23], and DRBA [24] also function exclusively in the post-deployment stage; they adjust reader activation or interrogation ranges but remain constrained by the initial random placement and by fixed-range assumptions. MR2D [25] applies a deterministic placement strategy with adjustable discrete ranges, but its candidate reader positions are limited to locations within each cluster, which may result in deploying more readers than necessary. Taken together, these comparisons highlight the need for a deterministic deployment framework that can provide predictable coverage and interference-aware performance in large-scale IIoT RFID systems.

In summary, previous research on RFID interference mitigation has primarily emphasized communication-level coordination or post-deployment optimization to reduce redundant readers and minimize interference. Although these methods can improve system performance under certain conditions, they generally rely on stochastic deployment or reactive adjustment, which may lead to incomplete coverage or unpredictable interference zones. Motivated by these limitations, the next section presents the system model and formulates the deterministic reader deployment problem addressed in this study.

## 3. System Model and Problem Formulation

Consider a set of RFID tags denoted by *T* = {*t*_1_, *t*_2_, *t*_3_, …, *t_n_*}. The tags are assumed to be randomly distributed over a two-dimensional plane of size *w* × *l*. The positions of all tags are known in advance, which can be obtained through localization techniques or Global Positioning System (GPS) data. The goal is to determine the optimal placement of RFID readers that can fully cover these tags while minimizing interference and the total number of readers required.

A deterministic deployment strategy is adopted for reader placement. Each reader *r_i_* ∈ *R* = {*r_i_*|1 ≤ *i* ≤ *m*, *m* = |*R*|} possesses an adjustable interrogation range denoted as *d_i_* ∈ *D* = {*d_i_*|1 ≤ *i* ≤ *k*, *k* = |*D*|}, where *D* represents the set of available reading ranges. For each reader, the appropriate reading range is selected according to the proposed algorithm in order to achieve efficient coverage and avoid interference. A tag *t_i_* is considered covered when at least one reader can successfully interrogate it; this condition is represented by a binary function *F*(*t_i_*), where *F*(*t_i_*) = 1 if *t_i_* is covered and *F*(*t_i_*) = 0 otherwise. The set of tags covered by reader *r_i_* is represented as *C*(*r_i_*).

The proposed system model is well suited for industrial and logistics IIoT environments, including smart factory production lines and warehouse management systems. In such settings, RFID tags are typically attached to products, pallets, or equipment, and the proposed algorithm provides the backend planning mechanism that determines optimal reader locations and corresponding interrogation ranges. This enables reliable full coverage, minimizes reader interference, and supports stable data acquisition for higher-level IIoT functions such as inventory tracking, automated material handling, and real-time monitoring.

The objective of this study is to minimize the total number of deployed readers while maintaining complete tag coverage and avoiding overlap among reader interrogation zones. The mathematical formulation of the proposed problem is defined through three core equations. Equation (1) expresses the objective of minimizing the total number of deployed readers required to achieve complete coverage of all tags within the monitored region. This objective reflects the efficiency of the deployment strategy in reducing both hardware costs and potential interference among readers. Equation (2) enforces the coverage constraint, ensuring that every tag in the set *T* is covered by at least one reader. This condition guarantees full accessibility of tag information across the monitored field and prevents coverage gaps that may lead to incomplete data collection. Equation (3) defines the interference avoidance constraint, which restricts overlapping tag coverage between any two active readers *r*_i_ and *r_j_*. This constraint is designed to prevent reader-to-tag interference, thereby maintaining reliable communication and reducing redundant interrogation of the same tag. Together, these three equations constitute a constrained optimization problem that seeks to achieve complete tag coverage with the minimum number of readers while preserving collision-free communication.

(1) $$\min\;\left|R\right|$$

(2) ∑Fti∈Tti =T

(3) ∑Cri∩Crjri,rj∈R=∅, ∀ri≠rj

## 4. The Concept and Approach

This section presents the design concept and operational procedure of the proposed Reader Deployment Algorithm with Adjustable Reader range (RDA2R). The algorithm aims to determine optimal reader positions and corresponding interrogation ranges based on the known spatial distribution of RFID tags. By integrating deterministic placement with adaptive range adjustment, the proposed framework effectively mitigates reader-to-tag interference and minimizes the number of deployed readers required for complete tag coverage.

### 4.1. Virtual Reader Initialization

To evaluate potential reader deployment locations, the monitored area is divided into a uniform grid structure. Each intersection point within this grid represents a candidate position for a virtual reader. For a two-dimensional field with dimensions *w* × *l* and a grid interval of one unit, the total number of virtual readers is (*w* − 1) × (*l* − 1). This grid-based initialization provides an evenly distributed set of reference points across the monitored region. These virtual readers serve as reference points for subsequent weight evaluation and actual reader deployment.

### 4.2. Evaluation of Tag Coverage Weights

Based on the initialized grid, each virtual reader is assigned a coverage weight according to the number of tags located within its maximum reading range. The weight reflects the potential contribution of that position to overall tag coverage. To prevent tag interference, a competitive assignment strategy is applied: each tag is temporarily associated with the nearest virtual reader based on Euclidean distance, ensuring that each tag contributes to only one reader’s weight. If a tag lies within the coverage of multiple readers, it is assigned to the reader with the smallest index. Tags outside all readers’ maximum ranges are excluded. This step establishes a weighted coverage map that will guide the subsequent reader selection process.

**Figure 2:**
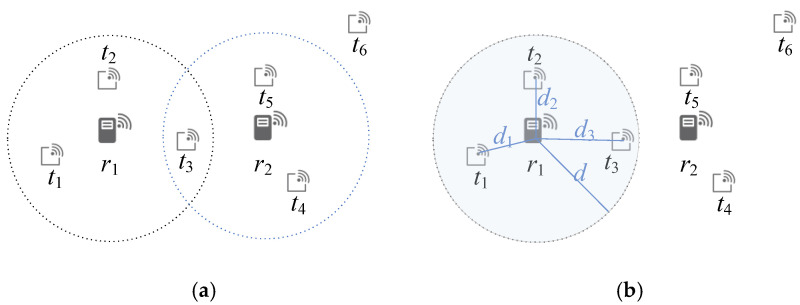
Conceptual illustration of the proposed RDA2R algorithm: (**a**) evaluation of tag-coverage weights for virtual readers, and (**b**) selection of reader deployment locations with interference avoidance and adaptive range adjustment, where each deployed reader refines its interrogation radius based on the farthest covered tag to minimize redundant overlap while maintaining full coverage.

As illustrated in Figure 2a, each tag temporarily selects the nearest reader as its potential connection. For example, tag *t*_2_ is closer to reader *r*_1_ than to *r*_2_, and thus contributes to *r*_1_’s weight. When multiple readers have equal distance to a tag, the one with the smaller index is prioritized. Tags located beyond the maximum reading range of all readers are excluded from the weight calculation. This weighting process establishes a preliminary estimation of each reader’s coverage effectiveness.

### 4.3. Reader Selection and Interference Handling

According to the computed coverage weights, virtual readers are ranked in descending order. The algorithm sequentially activates readers starting from the highest-weight candidate. For each candidate reader *r_i_*, it verifies whether its coverage overlaps with that of any already-activated reader. If no overlapping tags are detected, *r_i_* is deployed as an active reader. Otherwise, *r_i_* is skipped to avoid reader-to-tag interference, and the algorithm proceeds to the next candidate.

Figure 2b illustrates the weighted selection process. Reader *r*_1_ covers tags {*t*_1_, *t*_2_, *t*_3_} with a weight of three, while reader *r*_2_ covers tags {*t*_4_, *t*_5_} with a weight of two. Since *r*_1_ yields the highest weight and has no overlapping tags with other active readers, it is deployed first. In the following iteration, *r*_2_ is evaluated but rejected due to overlap with *r*_1_’s interrogation area. This iterative selection continues until no further readers can be deployed without interference.

### 4.4. Adaptive Range Adjustment

Initially, all readers are assumed to operate at the maximum allowable reading range. After the initial deployment, each active reader’s range is refined according to the farthest tag within its coverage. The interrogation radius is reduced to exactly reach that tag, thus minimizing redundant overlap while ensuring complete tag coverage. As illustrated in Figure 2b, reader *r*_1_ initially covers three tags {*t*_1_, *t*_2_, *t*_3_} with distances *d*_1_, *d*_2_, and *d*_3_, respectively. Since *d*_3_ is the largest, the reader’s final interrogation radius is set to *d*_3_.

After the initial range adjustment, the algorithm performs an iterative refinement in which the maximum allowable reading radius is reduced by one unit at each step. This unit decrement matches the spatial resolution of the discretized grid used for tag locations and candidate reader positions, ensuring that all feasible configurations are examined. For each reduced radius, the algorithm returns to Step 2 and executes Steps 2–4 to identify additional reader positions that satisfy the updated interference constraints. As the maximum radius becomes smaller, candidate readers are less likely to produce overlapping coverage, which helps suppress reader–tag interference. Larger radii may allow a single reader to cover more tags but often generate excessive overlap with neighboring readers. Smaller radii confine each reader’s coverage region and may expose feasible placements that were previously blocked by interference. By evaluating radii from the initial upper bound down to the minimum value of one unit, the algorithm ensures that all tags are covered in at least one iteration while progressively reducing redundant coverage and interference. This iterative reduction process produces a final deployment that achieves complete coverage with minimal overlap.

By integrating grid-based initialization, weighted tag assignment, interference-aware selection, and iterative range adjustment, the proposed RDA2R algorithm achieves efficient, interference-minimized RFID reader deployment while ensuring full tag coverage in IIoT environments.

### 4.5. Pseudocode of the Proposed RDA2R

The complete procedure of the proposed RDA2R algorithm is summarized in Algorithm 1. This pseudocode consolidates the individual components described in Section 4.1, Section 4.2, Section 4.3 and Section 4.4, including grid-based initialization, weighted tag assignment, interference-aware activation, and adaptive radius refinement.
**Algorithm 1:** Reader Deployment Algorithm with Adjustable Reader range (RDA2R)Input:    *T*: set of tags, each tag *t* ∈ *T* has coordinates (*x_t_*, *y_t_*)    *W*, *L*: width and length of the monitored field    *d_min_*: minimum allowable reading radius (one unit distance)    *d_max_*: maximum allowable reading radius    Δ: grid interval (one unit distance)  Output:    *R_act_*: set of deployed readers with positions and final radii // Step 1: Grid-based initialization    1: Generate all virtual reader positions *R_virt_* on a Δ-grid.    2: *R_act_* ← ∅    3: *d_cur_* ← *d_max_*
   4: while *d_cur_* ≥ *d_min_* do  // Step 2: Weighted Tag Assignment    5:    For each reader *r* ∈ *R_virt_*, rest its temporary coverage set and weight.    6:    For each tag *t* ∈ *T* do    7:      Find all readers within distance *d_cur_* of *t*.    8:      Assign *t* to the nearest reader that covers it.    9:    end for  10:    Compute coverage weight for each reader (number of assigned tags).  // Step 3: Interference-Aware Activation  11:    Sort readers in descending order of weight.  12:    For each reader *r* in sorted order do  13:      If *r* does not conflict with any activated reader then  // Step 4: Adaptive Range Adjustment  14:         Determine *r*’s final radius as the distance to its farthest assigned tag.  15:         Add *r* to *R_act_*.  16:      end if  17:    end for  // Iterative Reduction of Maximum Radius  18:    *d_cur_* ← *d_cur_* − 1  19: end while  20: return *R_act_*

### 4.6. Conceptual and Algorithmic Differences from MR2D [25] and DRBA [24]

The proposed RDA2R algorithm differs substantially from DRBA [24] and MR2D [25] in both conceptual design and deployment objectives. DRBA [24] is formulated as a post-deployment collision-avoidance mechanism in which all reader positions are fixed in advance. Its primary goal is to prevent reader–tag interference by selecting interrogation radii from a discrete candidate set. This objective can be expressed as:

(4) $$\max\;\left|\underset{r_{i}\in R}{\cup C\left(r_{i},d\right)}\right|\;s.t.\;C\left(r_{i},d\right)\cap C\left(r_{j},d\right)=\emptyset ,\;i\neq j.$$

DRBA [24] operates exclusively after deployment, adjusts only the interrogation ranges, and its performance is therefore constrained by the initial random placement and the discrete nature of its radius options.

In contrast, MR2D [25] formulates deployment as a deterministic optimization problem. It clusters spatially close tags into groups and uses the cluster centers as candidate reader locations. The placement objective can be written as:

(5) $$\min\;\left|R\right|\;s.t.\;\forall t_{j}\in T,\;\exists r_{i}\in R\;:\;t_{j}\in C\left(r_{i}\right).$$

However, MR2D [25] restricts reader placement to cluster centers and relies on a fixed set of discrete interrogation radii, which limits spatial flexibility and may lead to unnecessary readers when tag distributions are irregular or highly nonuniform.

RDA2R addresses these limitations by integrating grid-based deterministic placement, interference-aware reader activation, and adaptive range refinement. Unlike DRBA [24] and MR2D [25], RDA2R evaluates all feasible grid points as candidate reader locations rather than restricting them to precomputed cluster centers or an initial deployment. Each activated reader’s interrogation radius is adaptively set to the farthest assigned tag instead of being selected from a discrete set, enabling fine-grained range control. This integrated design ensures complete tag coverage while preventing reader-to-tag interference and minimizing the number of active readers. Consequently, RDA2R offers more spatial flexibility and provides a more effective deployment strategy than DRBA [24] and MR2D [25]. Table 2 summarizes these conceptual and algorithmic differences, including deployment assumptions, optimization goals, and range-design strategies.

## 5. Complexity Analysis

In this section, it is established that the problem discussed in this paper falls under the category of NP-hard. Furthermore, an analysis of the time complexity of the proposed algorithm is conducted.

### 5.1. NP-Hard Problem

To show the reader deployment problem discussed in this paper is NP-hard, the problem can be reduced to the set covering problem, which is an existing NP-hard problem [26,27]. The set covering problem is illustrated as follows.

***Instance:*** *Given a set U and a collection of its subsects denoted as S = s_1_* ∪ *s_2_* ∪ *… ∪ s_n_, where each subset s_i_ is a subset of U.*

***Question:***  *Find the minimum collection of subsets C, such that each element belongs to at least one subset in C.*

Next, the process of transforming the reader deployment problem into a set covering problem will be illustrated. Firstly, the set formed by the tags is defined as *U*, and for each virtual reader *r_i_*, the set of tags it can cover is defined as *s_i_*, where *S* = *s*_1_ ∪ *s*_2_ ∪ … ∪ *s_n_*, with *n* being the number of virtual readers in the target field, and *S* = *U*. In the process of selecting virtual readers, it is determined whether to deploy actual readers at those locations based on the magnitude of the weights assigned to *s_i_*. These weights are calculated based on the tags covered by the virtual reader. In other words, in addressing the reader deployment problem, the goal is also to find the minimum subset from the subsets of *S* whose union equals *U* (i.e., finding the minimum collection of subsets *C*, such that each element belongs to at least one subset in *C*). The distinction lies in the calculation and ordering of weight values, which can be accomplished in polynomial time. Since the reader deployment problem can be transformed into a set covering problem in polynomial time, the reader deployment problem is also NP-hard.

### 5.2. Complexity Analysis

To analyze the computational complexity of the proposed RDA2R algorithm, the notation used in this section is introduced as follows. Let *n_t_* denote the number of RFID tags in the monitored region. Let *n_r_* = (*l* − 1) × (*w* − 1) represent the number of virtual readers generated by a grid-based discretization of the field, where the grid interval is set to 1. During the execution of the algorithm, a subset of these virtual readers is activated; let *n_q_* denote the number of activated readers in a given iteration, where *n_q_* ≤ *n_r_*. For each activated reader, the number of tags temporarily assigned for determining its refined interrogation radius is denoted as *n_w_*, where *n_w_* ≤ *n_t_*. The algorithm evaluates every feasible interrogation radius between *d_max_* and *d_min_*, resulting in *z* = *d_max_* − *d_min_* + 1 iterations.

The major computational effort arises in Steps 2 through 4 of Algorithm 1. In Step 2, each virtual reader evaluates its coverage with respect to all tags, which leads to a time complexity of *O*(*n_r_n_t_*). Step 3 sorts the virtual readers according to their weights, requiring *O*(*n_r_*log*n_r_*). After sorting, each candidate reader is checked for potential interference with the activated readers. The worst-case cost of this process is *O*(*n_q_*^2^), and this value is bounded above by *O*(*n_r_*^2^) since *n_q_* never exceeds *n_r_*. Following activation, Step 4 refines the interrogation radius of each activated reader by examining the distances to its assigned tags. This refinement process requires *O*(*n_q_n_w_*) time and is therefore bounded above by *O*(*n_r_n_t_*), because *n_q_* ≤ *n_r_* and *n_w_* ≤ *n_t_*. By combining these components, the computational cost of one radius iteration can be expressed as

*O*(*n_r_n_t_*) + *O*(*n_r_*log*n_r_*) + *O*(*n_r_*^2^) + *O*(*n_r_n_t_*) (6)

which simplifies to

*O*(*n*_*r*_*n*_*t*_ + *n*_*r*_log*n*_r_ + *n*_*r*_^2^) (7)

Since the same procedure is repeated for each of the *z* considered interrogation radii, the total time complexity of the RDA2R algorithm becomes

*O*(*z*(*n*_*r*_*n*_*t*_ + *n*_*r*_log*n*_*r*_ + *n*_*r*_^2^)) (8)

When the monitored field size and the grid interval remain unchanged, the value of *n_r_* = (*l* − 1)(*w* − 1) does not scale with the number of tags. Under such conditions, nr can be regarded as a constant, and the above expression simplifies to

*O*(*n*_*t*_*z*) (9)

The simplified form applies only when the grid configuration remains fixed. In the general case, the complete expression *O*(*z*(*n_r_n_t_* + *n_r_*log*n_r_* + *n_r_*^2^)) describes the computational complexity of the RDA2R algorithm.

## 6. Performance Evaluation

### 6.1. Simulation Setup

In this section, the performance of the proposed RDA2R algorithm is evaluated and compared with two benchmark methods: ARLDL (Adjustable Reader with Limited Deployment Location) and MR2D [25]. ARLDL is a simplified variant derived from the RDA2R framework and is designed to examine the effects of deployment location constraints and fixed interrogation radii on reader coverage and interference behavior. In this method, readers are restricted to grid intersection points that contain tags, and the interrogation radius is limited to three discrete values of 1, 5, and 10 units of distance. These values represent abstract simulation units that correspond to the spatial resolution of the discretized grid used in the evaluation. This configuration enables analysis of how spatial deployment restrictions influence coverage efficiency, redundant reader activation, and the mitigation of interference. By comparing ARLDL and MR2D [25] with the proposed RDA2R, the evaluation highlights the advantages of adaptive range adjustment and deterministic placement in achieving complete coverage using fewer readers.

The simulation setting in this study follows the abstract grid-based framework adopted in MR2D [25]. The deployment field is defined as a 200 × 200 two-dimensional area populated with 100 to 500 RFID tags, and the interrogation radii, spatial coordinates, and reader–tag interactions are expressed using abstract simulation units that correspond to the spatial resolution of the grid. Readers are assumed to have adjustable interrogation ranges from 1 to 10 units with a step interval of 1 unit. This adjustable-range assumption is conceptually consistent with the tunable-range capabilities of commercially available long-range RFID readers such as those from Nundnet, which support configurable transmit power levels from 6 to 24 dBm and read-range profiles of 5 m, 10 m, 15 m, and 20 m [28]. These capabilities demonstrate the practical feasibility of employing tunable interrogation radii in IIoT environments. Similar to MR2D [25], the evaluation in this study does not model hardware-level characteristics such as antenna beamwidth, modulation schemes, reading cycles per second, specific tag chipsets, or link-budget values measured in dBm. Physical walls and other obstructions are also not included, and all experiments are conducted in a homogeneous two-dimensional environment. This simulation design isolates the algorithmic behavior of the evaluated methods and ensures a direct and fair comparison among RDA2R, ARLDL, and MR2D [25].

All simulations are implemented in Python 3.12 and executed on a workstation equipped with an Intel i7-11700 processor (2.50 GHz), 32 GB RAM, and a 64-bit Windows 10 operating system. Each experiment is independently repeated 100 times, and the averaged results are reported. The error bars shown in figures represent the standard deviation across these repeated simulation runs, allowing readers to observe the variability of each method. In this study, the grid resolution is fixed at Δ = 1 unit, which corresponds to the spatial discretization used for both tag locations and candidate reader positions. The range-adjustment step is also fixed at 1 unit to maintain consistency within the deployment model. For a fair comparison, each algorithm is evaluated under its original methodological assumptions. MR2D [25] uses a predefined discrete set of interrogation radii, and our implementation follows this specification to ensure faithful reproduction of the method. ARLDL, which examines fixed deployment locations and non-adaptive radii, is likewise evaluated using its predefined radius set. The proposed RDA2R algorithm differs in that it selects interrogation radii adaptively within the allowable range of 1 to 10 units, enabling a consistent comparison between adaptive and non-adaptive strategies. The performance evaluation focuses on three criteria: (1) the number of readers required to achieve full tag coverage, (2) the average number of tags covered per reader, and (3) the number of readers needed to reach different coverage levels. As this study relies on simulation-based analysis, physical validation using adjustable-range readers will be conducted in future work to further examine the practical behavior of tunable interrogation radii in real deployment environments.

### 6.2. Tag Distribution Scenarios

The experimental setup of MR2D [25] is referred to for consistency in simulation design, and two distinct tag placement patterns are considered to represent realistic deployment environments: random distribution and congregation distribution. In the random distribution scenario, all tags are uniformly and independently placed across the monitored field, ensuring an even spatial density. The congregation distribution scenario simulates environments where tags tend to cluster around certain locations. Specifically, 10% of the total tags are first placed uniformly at random across the field and serve as reference centers for subsequent placement. The next 70% of tags are then deployed within a localized region around these reference centers, restricted to a radius equivalent to 10% of the field size. For example, in a 200 × 200 (units of distance)^2^ two-dimensional field, this corresponds to a clustering radius of 20 units. The remaining 20% of tags are again distributed uniformly across the field to represent scattered or isolated objects.

### 6.3. Experiment 1: Comparison of the Number of Deployed Readers Under Different Tag Densities

**Figure 3:**
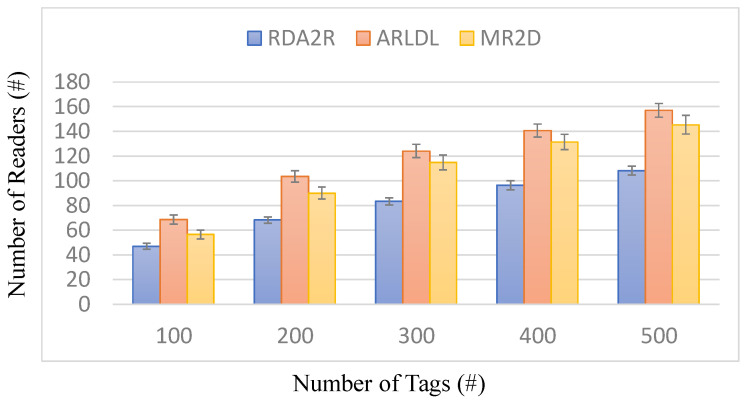
Number of deployed readers under different tag densities (random distribution). Error bars represent the standard deviation across all simulation runs.

**Figure 4:**
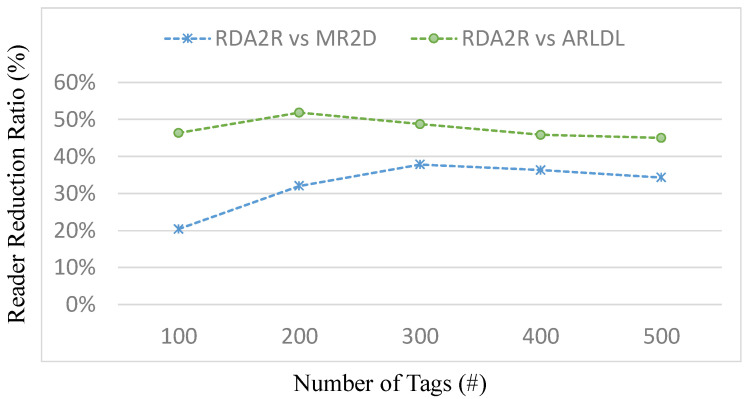
Reader reduction ratio under different tag densities (random distribution). The reader reduction ratio (%) is computed as (*X* − RDA2R)/RDA2R × 100%, where *X* denotes MR2D [25] or ARLDL.

**Figure 5:**
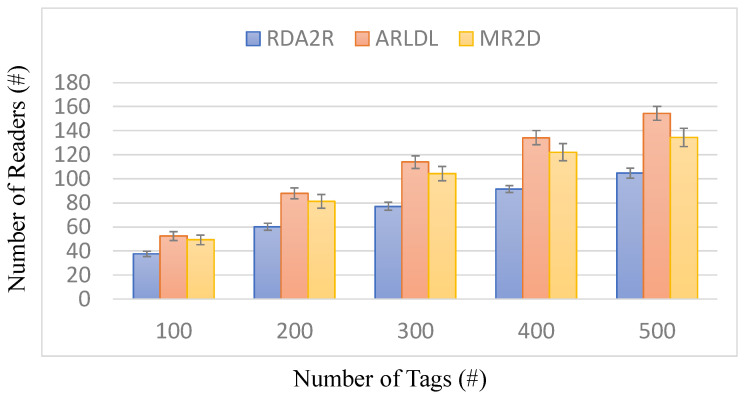
Number of deployed readers under different tag densities (congregation distribution). Error bars represent the standard deviation across all simulation runs.

**Figure 6:**
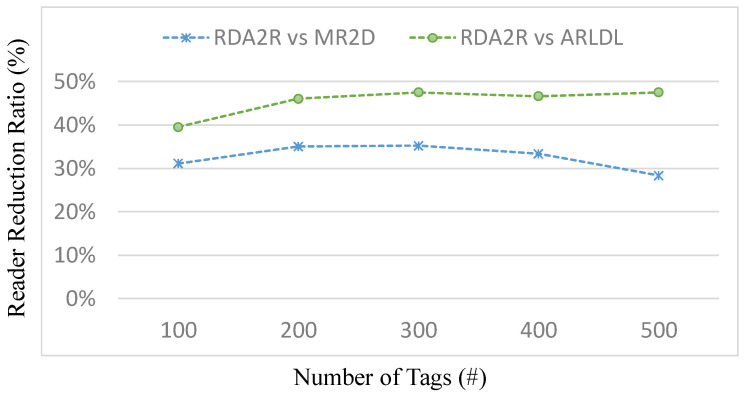
Reader reduction ratio under different tag densities (congregation distribution). The reader reduction ratio (%) is computed as (*X* − RDA2R)/RDA2R × 100%, where *X* denotes MR2D [25] or ARLDL.

Figure 3, Figure 4, Figure 5 and Figure 6 present the number of deployed readers required by the evaluated algorithms under varying tag densities for two tag placement patterns: random distribution (Figure 3 and Figure 4) and congregation distribution (Figure 5 and Figure 6). Figure 3 and Figure 5 report the required number of readers to achieve full tag coverage, while Figure 4 and Figure 6 show the corresponding reader-reduction ratios relative to ARLDL and MR2D [25]. In both scenarios, the proposed RDA2R algorithm consistently requires the fewest readers to achieve full tag coverage, and the improvement ratios shown in Figure 4 and Figure 6 quantify its relative advantage over ARLDL and MR2D [25]. Together, these four figures clearly demonstrate RDA2R’s overall deployment efficiency and its ability to scale gracefully as tag density increases.

Under the random distribution scenario (Figure 3 and Figure 4), RDA2R deploys between 46.93 and 108.27 readers as tag density increases from 100 to 500. In comparison, ARLDL requires 68.67–157.02 readers and MR2D [25] requires 56.51–145.39 readers. The improvement ratios further highlight this advantage: at 100 tags, RDA2R uses 20% fewer readers than MR2D [25] and 46% fewer than ARLDL; at 300 tags, the reductions reach 38% and 49%, respectively; and even at 500 tags, RDA2R still maintains reductions of 34% relative to MR2D [25] and 45% relative to ARLDL. These results indicate that RDA2R provides stable and efficient deployment in uniformly distributed environments.

For the congregation distribution scenario (Figure 5 and Figure 6), RDA2R maintains similarly strong performance. As tag density increases from 100 to 500, RDA2R deploys 37.59–104.68 readers, whereas ARLDL and MR2D [25] require 52.45–154.45 and 49.29–134.35 readers, respectively. At 100 tags, RDA2R achieves reductions of 40% relative to ARLDL and 31% relative to MR2D [25]. At 400 tags, the reductions remain substantial at 46% and 33%. These results show that RDA2R performs reliably even in clustered environments, where overlapping coverage regions and competition among nearby virtual readers often inflate reader counts for the baseline methods.

The performance of RDA2R results from its adaptive and interference-aware deployment mechanism. In random distributions, tag distances vary smoothly across the field, allowing the adaptive ranging mechanism to reduce each reader’s interrogation radius to the minimum required for its assigned tags. This minimizes unnecessary overlap and prevents excessive deployments, contributing significantly to the improvements observed in Figure 3 and Figure 4. In congregation distributions, candidate readers within clustered regions often compete to cover similar groups of tags, making interference control particularly important. RDA2R’s conflict-checking rule ensures that only non-overlapping readers are activated, thereby avoiding the redundant placements frequently observed in ARLDL and MR2D [25] and explaining the gains shown in Figure 5 and Figure 6.

Overall, Figure 3, Figure 4, Figure 5 and Figure 6 collectively demonstrate that RDA2R offers the most efficient and interference-free reader deployment across both uniform and clustered spatial environments. The algorithm’s consistent reductions in reader count and its adaptability to different tag distributions confirm its robustness and scalability for large-scale RFID-enabled IIoT applications.

### 6.4. Experiment 2: Average Tag Coverage per Reader

**Figure 7:**
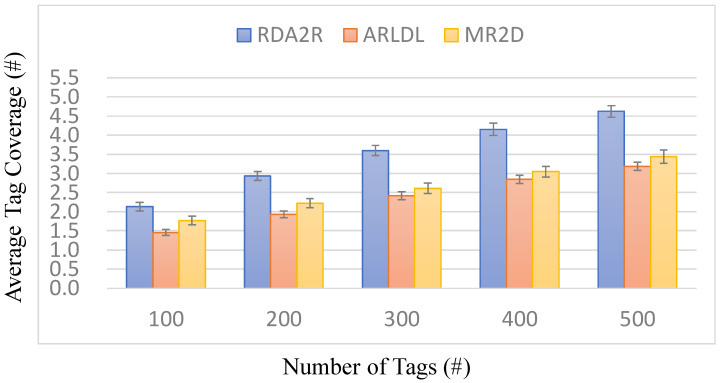
Average tag coverage per reader under different tag densities (random distribution). Error bars represent the standard deviation across all simulation runs.

**Figure 8:**
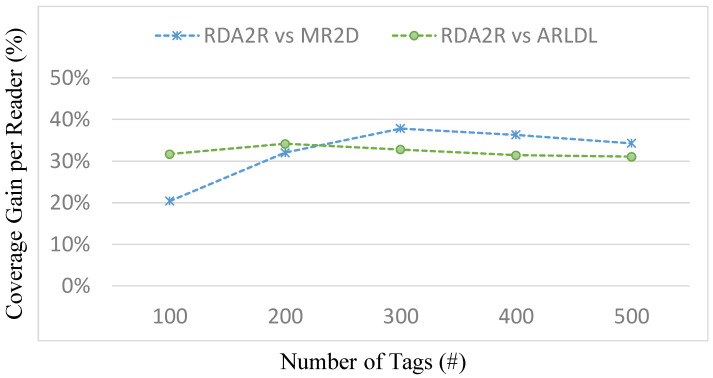
Coverage gain per reader under different tag densities (random distribution). The coverage gain per reader (%) is computed as (*X* − RDA2R)/RDA2R × 100%, where *X* denotes MR2D [25] or ARLDL.

**Figure 9:**
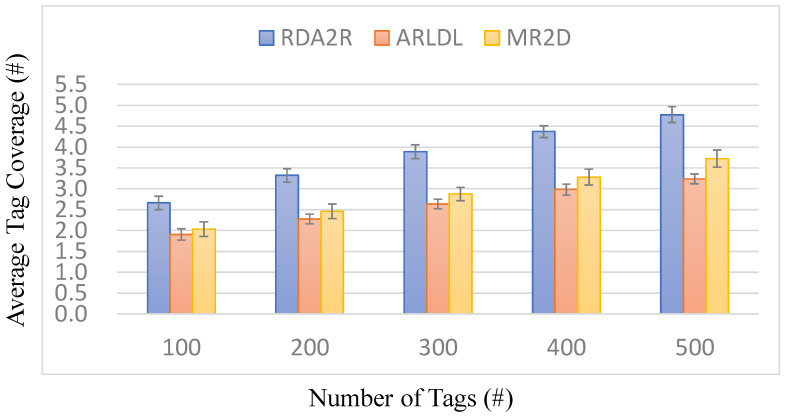
Average tag coverage per reader under different tag densities (congregation distribution).

**Figure 10:**
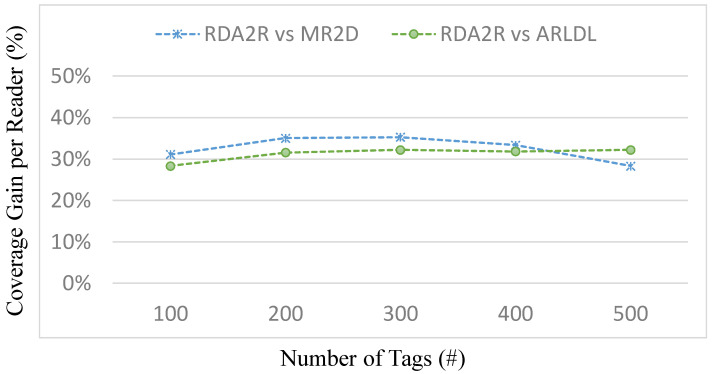
Coverage gain per reader under different tag densities (congregation distribution). The coverage gain per reader (%) is computed as (*X* − RDA2R)/RDA2R × 100%, where *X* denotes MR2D [25] or ARLDL.

Figure 7, Figure 8, Figure 9 and Figure 10 evaluate the average number of tags covered by each deployed reader under varying tag densities for both random and congregation distributions. Figure 7 and Figure 9 show the average number of tags covered per reader, while Figure 8 and Figure 10 present the corresponding coverage gains relative to ARLDL and MR2D [25]. Across all four figures, the proposed RDA2R algorithm consistently achieves the highest tag coverage per reader, demonstrating its ability to utilize each deployed reader more efficiently than the baseline methods.

Under the random distribution scenario (Figure 7 and Figure 8), RDA2R exhibits a steady increase in average tag coverage as tag density grows from 100 to 500. Specifically, RDA2R’s per-reader coverage increases from 2.13 to 4.62 tags, outperforming ARLDL, which increases only from 1.46 to 3.18, and MR2D [25], which increases from 1.77 to 3.44. The coverage gain results in Figure 8 further highlight this advantage: RDA2R improves per-reader coverage by an average of 32.21% over ARLDL and 32.17% over MR2D [25] across all tested densities. These results demonstrate that RDA2R maximizes each reader’s effective contribution to coverage in uniformly distributed environments, leading to consistently higher utilization efficiency.

In the congregation distribution scenario (Figure 9 and Figure 10), RDA2R maintains similarly performance. As tag density increases from 100 to 500, RDA2R achieves per-reader coverage values of 2.66, 3.32, 3.89, 4.37, and 4.78. ARLDL covers only 1.91 to 3.24 tags per reader, and MR2D [25] covers 2.03 to 3.72. The corresponding coverage gains shown in Figure 10 indicate improvements of approximately 31% over ARLDL and 33% over MR2D [25]. These results confirm that RDA2R effectively allocates tags among readers even in clustered environments, where overlapping interrogation zones often reduce the effective coverage achieved by the baseline algorithms.

The performance of RDA2R in this experiment is driven primarily by its iterative range adjustment and competitive tag-to-reader assignment mechanisms. In both random and congregation distributions, RDA2R dynamically reduces each reader’s interrogation radius to match the distance of its farthest assigned tag. This prevents redundant overlap and ensures that each deployed reader is responsible only for the tags most relevant to its position. At the same time, the competitive assignment rule ensures that each tag is exclusively associated with the nearest feasible reader, eliminating duplicate associations and improving spatial allocation efficiency. These combined mechanisms explain why RDA2R consistently achieves the highest per-reader coverage across all densities and both spatial distributions.

Overall, Figure 7, Figure 8, Figure 9 and Figure 10 demonstrate that RDA2R not only minimizes the total number of required readers but also maximizes the effectiveness of each reader that is deployed. This dual efficiency underscores the scalability and practicality of RDA2R for large-scale RFID deployments, where both coverage completeness and hardware utilization are critical concerns.

### 6.5. Experiment 3: Number of Readers Required for Different Coverage Levels

**Figure 11:**
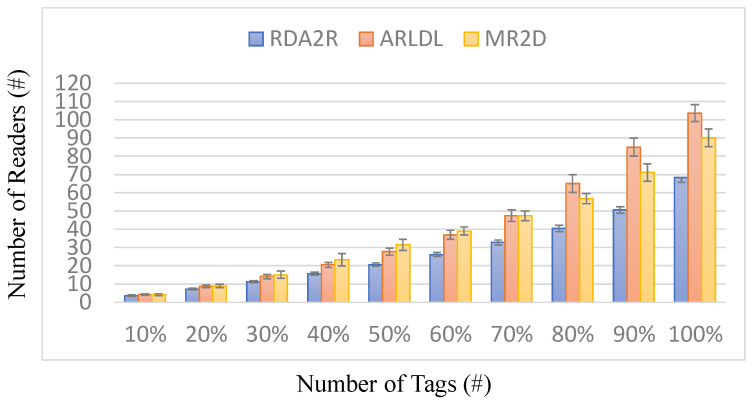
Number of readers required to achieve different coverage levels (random distribution). Error bars represent the standard deviation across all simulation runs.

**Figure 12:**
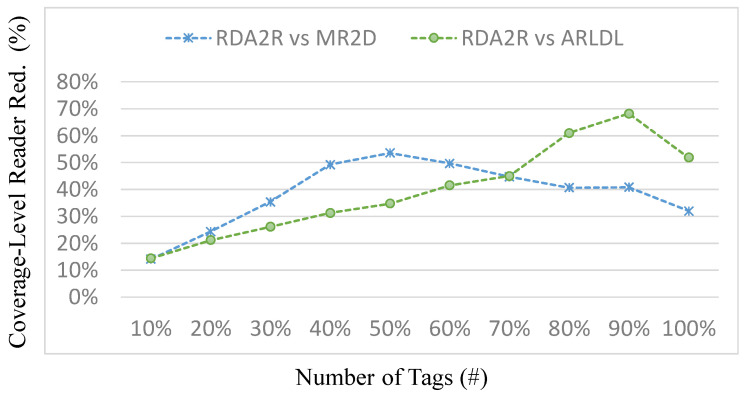
Reader requirement reduction to achieve different coverage levels (random distribution). The coverage-level reader reduction (%) is computed as (*X* − RDA2R)/RDA2R × 100%, where *X* denotes MR2D [25] or ARLDL.

**Figure 13:**
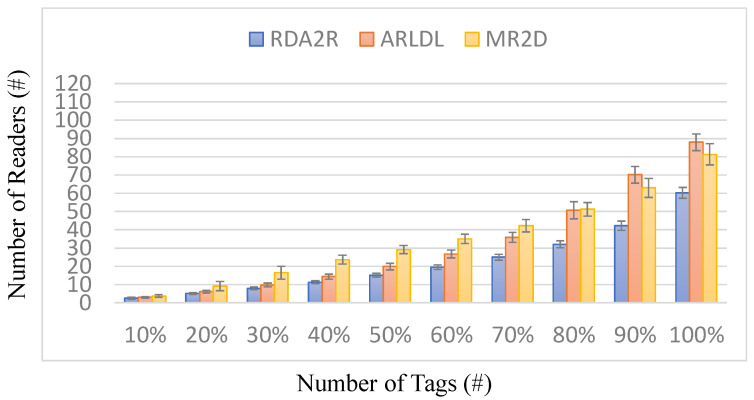
Number of readers required to achieve different coverage levels (congregation distribution). Error bars represent the standard deviation across all simulation runs.

**Figure 14:**
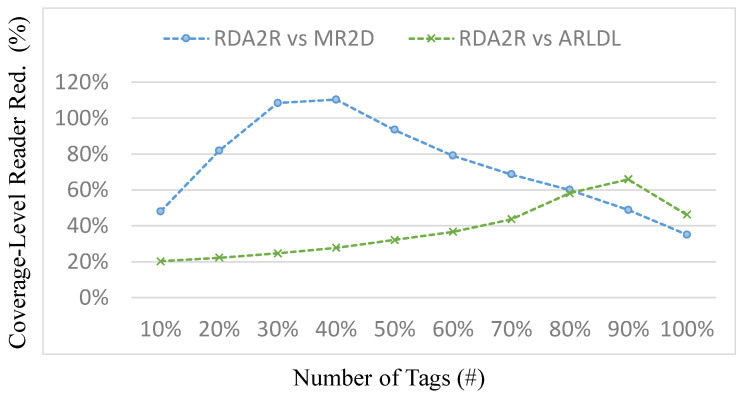
Reader requirement reduction to achieve different coverage levels (congregation distribution). The coverage-level reader reduction (%) is computed as (*X* − RDA2R)/RDA2R × 100%, where *X* denotes MR2D [25] or ARLDL.

Figure 11, Figure 12, Figure 13 and Figure 14 present the number of readers required by each algorithm to achieve partial coverage levels. In this experiment, the coverage requirement is defined on a normalized 0–100% scale, and the results are reported at 10% increments (i.e., 10% to 100%), which is a standard way to illustrate how reader demand varies as the target coverage level increases. Figure 11 and Figure 13 show the number of required readers under random and congregation distributions, respectively, while Figure 12 and Figure 14 show the corresponding coverage-level reader-reduction ratios relative to ARLDL and MR2D [25]. Across all conditions, RDA2R consistently requires the fewest readers to reach any given coverage target, demonstrating its scalability and efficiency across both uniform and clustered tag placements.

Under the random distribution scenario (Figure 11 and Figure 12), RDA2R gradually increases its reader count from 3.62 at 10% coverage to 68.22 at full coverage, while ARLDL requires 4.14–103.58 readers and MR2D [25] requires 4.13–90.06 readers. The improvement ratios in Figure 12 show that RDA2R achieves reductions of 14–54% relative to ARLDL and 14–68% relative to MR2D [25] across all coverage levels. Notably, even at 100% coverage, RDA2R still reduces reader usage by 52% compared with ARLDL and 32% compared with MR2D [25]. These results indicate that RDA2R maintains a clear advantage not only at full coverage but also at low and mid-range coverage levels, confirming its adaptability to varying coverage requirements.

In the congregation distribution scenario (Figure 13 and Figure 14), RDA2R again demonstrates strong and consistent performance. Its reader count increases from 2.48 at 10% coverage to 60.18 at 100% coverage. Under the same conditions, ARLDL requires 2.98 to 87.92 readers, and MR2D [25] requires 3.67 to 81.28. The reduction ratios in Figure 14 indicate improvements of up to 110% over MR2D [25] at 40% coverage and 46% and 35% reductions over ARLDL and MR2D [25], respectively, at full coverage. These results confirm that RDA2R remains robust even in clustered conditions, where overlapping coverage regions often cause excessive and inefficient deployments for the baseline algorithms.

The performance of RDA2R in achieving different coverage levels stems from its ability to identify reader placements that minimize interference while maximizing spatial efficiency. At low coverage levels, RDA2R preferentially activates high-weight candidate readers that naturally cover large tag groups without conflict. As coverage requirements increase, RDA2R’s interference-aware selection and adaptive range adjustment become increasingly important, preventing redundant overlap and maintaining efficient expansion of coverage without excessive reader activation. In clustered environments, the interference-avoidance component is particularly beneficial, as it prevents multiple readers from redundantly covering the same localized tag cluster, explaining the consistently high reduction ratios observed in Figure 13 and Figure 14.

Overall, Figure 11, Figure 12, Figure 13 and Figure 14 demonstrate that RDA2R not only reduces the number of readers required for full coverage but also scales effectively across partial coverage requirements. This makes RDA2R suitable for practical IIoT deployments where coverage goals may vary, and where minimizing hardware cost and interference is essential. The consistent improvements across both tag distributions confirm its robustness and flexibility in diverse spatial environments.

## 7. Conclusions

This paper presented RDA2R, a deterministic and interference-aware reader deployment framework for RFID-enabled IIoT environments. The proposed method integrates grid-based initialization, competitive tag-to-reader association, interference-aware reader activation, and adaptive range adjustment to achieve complete tag coverage with the minimum number of readers while suppressing redundant overlap and communication interference. Extensive simulations under random and congregation tag distributions confirm the robustness and scalability of RDA2R across different tag densities ranging from 100 to 500. Under the random tag distribution, RDA2R demonstrates stable scalability and balanced deployment efficiency as tag density increases. For full coverage, RDA2R reduces the number of deployed readers by approximately 46% compared with ARLDL and 32% compared with MR2D [25]. It also enhances tag-to-reader utilization by about 31–33%, indicating that each reader contributes more effectively to overall coverage while maintaining interference-free operation. Under the congregation tag distribution, where tags are spatially clustered, RDA2R exhibits stronger performance gains. The number of required readers decreases by roughly 47% relative to ARLDL and 33% relative to MR2D [25] at full coverage. When the partial coverage levels range from 10% to 100%, RDA2R consistently requires fewer readers than the benchmark methods. At an 80% coverage level, RDA2R achieves improvement ratios of approximately 58% over ARLDL and 60% over MR2D [25], indicating that it requires about one-third fewer readers to reach the same coverage. Even at full coverage, RDA2R maintains reductions of approximately 46% compared with ARLDL and 35% compared with MR2D [25], demonstrating its efficiency and scalability under clustered deployment conditions. Overall, RDA2R achieves reliable, scalable, and interference-minimized reader deployment for RFID-based IIoT applications. It ensures complete tag accessibility while significantly reducing hardware requirements and potential interference zones compared with benchmark algorithms. As this study is based on simulation, future work will include hardware validation using adjustable-range RFID readers to examine the practical deployment behavior of RDA2R in real IIoT environments.

## Figures and Tables

**Table 1:** Summary of strengths and limitations of existing RFID schemes.

Scheme	Category	Strengths	Limitations
DFSA [18]	Tag-to-tag	Reduces idle and collision slots through dynamic frame adjustment.	Addresses only tag-level timing; does not mitigate reader-level interference.
ILCM [19]	Tag-to-tag	Provides faster and more efficient tag-population estimation with lower computational cost.	Still dependent on estimation-based frame adjustment; does not address reader-level interference.
DPC [20]	Reader-to-reader	Mitigates mutual interference via power tuning.	Post-deployment only; reader positions fixed; no guarantee of complete coverage.
CSMA [21]	Reader-to-reader	Reduces contention among readers through carrier sensing and channel monitoring.	Post-deployment only; sensing overhead; cannot guarantee collision-free operation; ineffective in dense deployments.
NCD [22]	Reader-to-tag	Suppresses interference by deactivating redundant readers.	Post-deployment only; depends on initial random placement; static ranges.
NCDMD [22]	Reader-to-tag	Extends NCD [22] by incorporating motion detection to avoid unnecessary tag write operations in dynamic environments.	Post-deployment only; inherits NCD’s dependency on random initialization and fixed-range assumptions.
TSA [23]	Reader-to-tag	Threshold-based activation; TSAMD supports basic mobility.	Post-deployment only; threshold tuning required; coverage completeness not guaranteed.
DRBA [24]	Reader-to-tag	Adjusts interrogation ranges to reduce redundant overlap.	Post-deployment only; cannot relocate readers; dependent on initial deployment.
MR2D [25]	Reader-to-tag	Deterministic placement and supports adjustable ranges selected from a predefined discrete set.	Candidate locations restricted to positions within each cluster; may require more readers.

**Table 2:** Comparison of DRBA [24], MR2D [25] and RDA2R.

Scheme	Objective	Reader Locations and Deployment Capability	Range Design
DRBA [24]	Maximize the number of readable tags.	Readers are pre-deployed at fixed positions and cannot be relocated.	Uses a predefined discrete set of radii.
MR2D [25]	Minimize the number of readers.	Performs deterministic placement but candidate locations are restricted to cluster-derived positions.	Uses a predefined discrete set of radii.
RDA2R	Minimize the number of readers.	Performs deterministic grid-based placement across the entire monitored region.	Uses an adaptive and continuous radius.

## Data Availability

The simulation scripts and parameter configurations used in this study are openly available in the RDA2R GitHub repository at https://github.com/mcnlabcs/RDA2R (version v1.0, accessed on 28 November 2025).

## References

[1] Ando K., Kushihashi M., Kawaguchi H., Izumi S. (2025). Flexible Optical Tactile Force Sensor to Conduct Measurements from the Back of the Hand. IEEE Sens. J..

[2] Cheng C.-F., Wang C.-C., Chang H.-Y. (2024). An Automatic Cascaded Movement Approach to Solve the Energy Replenishment Problem in WPT-Based Mobile WRSNs. IEEE Trans. Autom. Sci. Eng..

[3] Djehaiche R., Aidel S., Sawalmeh A., Saeed N., Alenezi A.H. (2023). Adaptive Control of IoT/M2M Devices in Smart Buildings Using Heterogeneous Wireless Networks. IEEE Sens. J..

[4] Wang G., Yang J., Wei Y., Wang C., Li K., Feng C. (2025). Deep-Reinforcement-Learning-Driven Patient State Analysis and Resource Management in Near-Field IoE Healthcare Networks. IEEE Internet Things J..

[5] Cheng C.-F., Chen Y.-C., Lin J.C.-W. (2020). A Carrier-Based Sensor Deployment Algorithm for Perception Layer in the IoT Architecture. IEEE Sens. J..

[6] Herrera J.L., Galán-Jimnez J., Garcia-Alonso J., Berrocal J., Murillo J.M. (2023). Joint Optimization of Response Time and Deployment Cost in Next-Gen IoT Applications. IEEE Internet Things J..

[7] Qiu G., Tao W., Hwang R.-C., Xie C. (2025). Wide-Area Visual Monitoring System Based on NB-IoT. Sensors.

[8] Chithaluru P., Al-Turjman F., Kumar M., Stephan T. (2023). Computational-Intelligence-Inspired Adaptive Opportunistic Clustering Approach for Industrial IoT Networks. IEEE Internet Things J..

[9] Li T., Ma Y., Endoh T. (2023). From Algorithm to Module: Adaptive and Energy-Efficient Quantization Method for Edge Artificial Intelligence in IoT Society. IEEE Trans. Ind. Inform..

[10] Reis M.J.C.S. (2025). Lightweight Signal Processing and Edge AI for Real-Time Anomaly Detection in IoT Sensor Networks. Sensors.

[11] Bugshan N., Khalil I., Rahman M.S., Atiquzzaman M., Yi X., Badsha S. (2023). Toward Trustworthy and Privacy-Preserving Federated Deep Learning Service Framework for Industrial Internet of Things. IEEE Trans. Ind. Inform..

[12] Joha M.I., Rahman M.M., Nazim M.S., Jang Y.M. (2024). A Secure IIoT Environment that Integrates AI-driven Real-Time Short-Term Active and Reactive Load Forecasting with Anomaly Detection: A Real-World Application. Sensors.

[13] Liu Y., Chi C., Zhang Y., Tang T. (2022). Identification and Resolution for Industrial Internet: Architecture and Key Technology. IEEE Internet Things J..

[14] Meng Z., Liu Y., Gao N., Zhang Z., Wu Z., Gray J. (2021). Radio Frequency Identification and Sensing: Integration of Wireless Powering, Sensing, and Communication for IIoT Innovations. IEEE Commun. Mag..

[15] Xu L.D., He W., Li S. (2014). Internet of Things in Industries: A Survey. IEEE Trans. Ind. Inform..

[16] Kim D.-Y., Yoon H.-G., Jang B.-J., Yook J.-G. (2009). Effects of Reader-to-Reader Interference on the UHF RFID Interrogation Range. IEEE Trans. Ind. Electron..

[17] Cheng C.-F., Liao B.-Y. An Enhanced Approach for RFID Reader Deployment in Industrial IoT Systems with Collision Avoidance and Optimized Coverage. Proceedings of the IEEE DSA 2023.

[18] Keat C.S., Shyan L.N. Dynamic framed slotted ALOHA algorithm for RFID systems with enhanced tag estimation technique. Proceedings of the IEEE RFID-TA 13.

[19] Šolić P., Radić J., Rožić N. (2014). Energy Efficient Tag Estimation Method for ALOHA-Based RFID Systems. IEEE Sens. J..

[20] Bai Y., Xuan X.-W., Teng J.-F., Zhang L.-Y. A RFID Anti-Collision Algorithm Based on Distributed Power Control. Proceedings of the WiCOM 10.

[21] Birari S., Iyer S. Mitigating the reader collision problem in RFID networks with mobile readers. Proceedings of the IEEE ICON’05.

[22] Ma M., Wang P., Chu C.-H. A novel distributed algorithm for redundant reader elimination in RFID networks. Proceedings of the IEEE RFID-TA 13.

[23] Ma M., Wang P., Chu C.-H. (2018). Redundant Reader Elimination in Large-Scale Distributed RFID Networks. IEEE Internet Things J..

[24] Nguyen N.-T., Liu B.-H., Pham V.-T. A dynamic-range-based algorithm for reader-tag collision avoidance deployment in RFID networks. Proceedings of the ICEIC 2016.

[25] Wang Y.-C., Liu S.-J. (2017). Minimum-cost deployment of adjustable readers to provide complete coverage of tags in RFID systems. J. Syst. Softw..

[26] Agathos S., Papapetrou E. (2013). On the Set Cover Problem for Broadcasting in Wireless Ad Hoc Networks. IEEE Commun. Lett..

[27] Ke W.-C., Liu B.-H., Tsai M.-J. (2007). Constructing a Wireless Sensor Network to Fully Cover Critical Grids by Deploying Minimum Sensors on Grid Points Is NP-Complete. IEEE Trans. Comput..

[28] Long Range RFID Readers. https://nundnet.com/solutions/uhf-long-range-rfid-reader/.

